# The Cortical Topography of Visual Evoked Potentials Elicited by Chromatic and Luminance Motion

**DOI:** 10.2174/1874364100701010025

**Published:** 2007-12-17

**Authors:** E.G Laviers, M.P Burton, D.J McKeefry

**Affiliations:** Vision Science Research Group, School of Life Sciences, University of Bradford, Richmond Road, Bradford BD7 1DP, UK

**Keywords:** Colour, motion, visual evoked potentials (VEPs).

## Abstract

When motion-onset VEPs are elicited by moving luminance patterns, the motion specific component of the response, N2, is more prominent at electrode sites that overlay the lateral occipito-parietal cortex close to area V5/MT, than over the primary visual cortex. Functional segregation suggests that colour and motion processing should take place along different ventral occipito-temporal and lateral occipito-parietal pathways, respectively. Hence, a different topographical distribution might be expected for the motion-onset VEPs elicited by chromatic and luminance motion stimuli. We recorded motion-onset VEPs to moving luminance or isoluminant chromatic sinusoidal grating stimuli from five electrodes sites located at Oz, and at four locations (T1-T4) lateral to Oz, at intervals of 5% of the head circumference. Responses were recorded from 6 subjects over a range of speeds and contrasts. The results showed that the N2 component was maximal at similar lateral electrode locations (T2) for both luminance-defined and chromatically-defined motion. The earlier P1 component was of greatest magnitude at the occipital pole (Oz) and decreased with more lateral electrode placement and again this was the same for colour and luminance responses. These similarities suggest a common origin for VEPs elicited by colour and luminance defined motion.

## INTRODUCTION

Visual evoked potentials (VEPs) elicited by the onset of motion generate characteristic and highly repeatable waveforms in human subjects, which have an early positive wave (P1) with a latency of between 100-130 ms, followed by a negative wave (N2) at 160 - 200 ms [1-12]. The general consensus is that the N2 component reflects motion specific brain activity due to the fact that, amongst other properties, it is susceptible to directionally specific motion adaptation [13,14]. P1, on the other hand is considered to reflect pattern or luminance processing [4,6,8]. Analysis of the topographical variation of these components across the human scalp has further emphasised this dichotomy. A number of studies have demonstrated the fact that N2 amplitude is greatest at lateral electrode sites that are located over the occipitoparietal cortex, whilst P1 is predominant at recording sites over the primary visual cortex at the occipital pole [6,8,15-17]. The predominance of N2 at lateral recording sites presumably reflects proximity to the motion processing centre of the human brain, area V5/MT, which is located on the lateral aspect of the occipito-parietal cortex and is considered to be the source of the N2 component in the motion-onset VEP [18-21].

The foregoing studies have largely concentrated on motion-onset VEPs elicited by luminance stimuli, and whilst moving isoluminant chromatic patterns have been shown capable of eliciting motion specific responses from the human brain [22-23], little is known about the topographical distribution of responses generated by such stimuli. How and where the visual system processes the motion of purely chromatic stimuli is a question that continues to be a focus of interest in vision science. Earlier views, encapsulated by the doctrine of functional segregation in its strictest sense, carried with it the implication that the processing of colour and motion information took place along separate, segregated anatomical pathways [24]. Human brain imaging studies to a certain extent support this view, revealing the presence of functionally specialised areas for colour processing in the ventral occipito-temporal cortex [25], and for motion in the lateral occipito-parietal cortex [19]. Such segregation of function might lead to the expectation that there would be differences in the topographical distribution of VEPs generated by the onset of chromatic and luminance motion. However, the notion of a strict segregation between the processing of colour and motion has been called into question by numerous psychophysical results that clearly point to interactions between colour and luminance processing [26-30]. Moreover, on a physiological level it is clear that area V5/MT receives extensive input from the chromatic system [31-34] and is able in its own right to process motion signals that derive from moving coloured patterns, thus, raising the possibility that colour and motion are processed along identical anatomical pathways [34-36]. More recent ideas regarding chromatic motion processing suggest that particular parameters are important in determining how such stimuli are processed. For example, the analysis of high speed or high contrast chromatic stimuli is thought to be performed by low-level, motion energy detectors, similar to those involved in luminance motion processing. Low speed, low contrast chromatic motion stimuli, on the other hand, are more likely to be processed by higher-order or ‘feature tracking’ mechanisms [37-41].

The aim of this study is to examine the topographical distribution of VEPs elicited by the onset of chromatic motion across the human scalp, in an attempt to discover whether the components of such responses have a similar or different distribution to those generated by luminance motion stimuli. A similarity in their distributions would strongly suggest that the responses are derived from similar cortical areas and that the processing of colour and luminance motion share similar anatomical pathways. In addition, we were also interested in whether the topographical distribution of the chromatic motion onset VEP differed for fast versus slow speeds or high versus low contrasts, in the light of the proposals that different pathways might be involved in the analysis of these different kinds of motion stimuli.

## METHODS

### VEP Recording

VEPs were recorded from five active silver-silver chloride electrodes attached to the scalp placed at Oz (located above the inion by 10% of the inion-nasion distance) and at intervals of 5% head circumference to the left of Oz. The distribution of electrodes was based on the 10/20 system described by Jasper [42] although for ease of reference, the labelling of the four lateral electrodes was modified to T1 - T4, with T1 lying 5% of the head circumference lateral to Oz, electrode T2 lying 10% more laterally and so on (see Fig. (**1a**)). The ground electrode was placed on the forehead and linked ears were used as the reference. Electrode impedances were maintained below 5kΩ. Recording would continue until the response average showed little change in waveform, however, a minimum of 128 sweep averages were always recorded. A CED 1401 ‘micro’ and accompanying Signal software were used to average the VEPs. Amplifier (CED 1902) bandwidth was 0.5-30 Hz and sampling rate was 250 Hz over 1.496s. All stimuli were viewed binocularly at 114cm in a darkened room.

Fig. (**1b**) shows typical motion-onset VEPs recorded from the different electrode locations for one of the observers (VA). At Oz the motion-onset response has a characteristic waveform with three main peaks P1, N2 and P2 which vary in predominance depending upon the type of motion and recording site [11,12]. The amplitudes of these major components of the motion-onset waveform were measured peak-to-trough (i.e. P1 = N1-P1 and N2 = N2-P2). Peak-to-trough amplitude measurements have the advantage of not requiring the calculation of an amplitude baseline, which will be influenced by waveform components that are often governed by factors other than those desired for investigation.

### Stimuli

Motion-onset stimuli were generated using a VSG2/3 graphics card (version 5, Cambridge Research Systems) and presented on a Sony 21” FD Trinitron CRT (cathode ray tube) monitor. Calibration of stimuli was performed using a PR650 Spectrascan SpectraColorimeter. The stimuli took the form of sinusoidal, vertically oriented gratings with a spatial frequency of 1cycle/degree presented in a circular window of diameter equal to 7^o^. They were presented on a grey background (illuminant C x = 0.310, y = 0.316) of the same mean luminance, 25 cd/m^2^. A stimulus of 7^o^ was used to minimise luminance artefacts, which can occur due to chromatic aberration and changes in isoluminance with retinal eccentricity. It has been shown that luminance artefacts in isoluminant chromatic gratings can be minimised by using stimuli containing less than eight cycles [43]. The duty cycle consisted of the grating drifting to the left for 100msec and then remaining stationary for a further 650msec during which the observers maintained fixation on a centrally positioned cross.

Chromatic and achromatic versions of the grating stimuli were generated by modulation along the cardinal (L+M (black-white), L/M (red-green) and S/(L+M) (blue-yellow)) axes of DKL colour space [44]. In this space chromaticity varies with the angle of projection in the isoluminant plane (azimuth, ø) and luminance contrast varies with the angle of elevation out of the isoluminant plane (elevation, θ) with the isoluminant plane midway on the luminance axis. Along the ø = 0-180 axis S-cone excitation is minimised resulting in stimulation of only the L- and M-cones, conversely, along the ø = 90-270 axis L- and M-cone excitation is minimised leading to stimulation of the S-cones only. Isoluminance for the chromatic stimuli were determined for each subject by heterochromatic flicker photometry and minimum motion methods [45]. Isoluminance, as indicated by a minimum in the perception of flicker and/or motion, occurs at a particular luminance ratio:

1 $${\text{L}}_{\lambda 1}/\left({\text{L}}_{\lambda 1}+{\text{L}}_{\lambda 2}\right)$$

where L_λ1_ and L_λ2 _are the luminances of the two constituent colours of the chromatic grating stimulus. Photometric isoluminance corresponds to a luminance ratio of 0.5, however, across the four observers the subjectively measured isoluminant points varied between 0.47 - 0.51 for the L/M and S/(L+M) grating stimuli. By shifting the luminance ratios of the stimuli away from isoluminance by up to +/- 0.1 we could examine the effects of introducing small amounts of luminance contrast on the chromatic motion-onset VEP.

Motion-onset VEPs were elicited by achromatic and chromatic stimuli of different speeds ranging from a maximum of 10 deg/sec down to a minimum of 1 deg/sec. For achromatic or monochromatic stimuli standard measures of luminance contrast are acceptable as the three cone classes are modulated by the same amount. The component colours of heterochromatic stimuli, however, are not modulated by the same amount. For example, a chromatic L/M grating of high contrast will only modulate the L and M cones by a small amount as each cone type is similarly sensitive to the red and green components due to the large overlap in spectral sensitivity of the two cone classes. Cone contrast values were therefore computed for the chromatic stimuli to allow a closer comparison of the achromatic and chromatic results. The cone contrasts of the chromatic stimuli were calculated using the Judd modified values in order to compensate for errors in the short wavelength region of the CIE V(λ) function. X’, Y’, Z’ tristimulus values were then used in conjunction with cone fundamentals [46,47] to ascertain the degree of cone excitation for each component colour from which modulation of quantum catch (i.e. Michelson contrast) for each cone (Lc, Mc, Sc) was calculated. The square root of the sum of the squared cone contrast values (C, equation 1) was then computed to provide a single contrast value for the L/M and S/(L+M) axes.

2 C = √(Lc^2^+Mc^2^+Sc^2^)

### Observers

Six observers participated in the VEP experiments (mean age = 30; S.D. = 5.13). All subjects had visual acuities of 6/6 or better (with refractive correction where necessary). All subjects had normal colour vision according to the Farnsworth-Munsell 100 Hue test.

## RESULTS

### Topographical Variation of the Motion-Onset VEP as a Function of Stimulus Speed

Psychophysical studies of motion perception have led to the suggestion that different mechanisms exist for the processing of moving chromatic stimuli at low and high speeds. It has been proposed that slow moving (< 4 deg/s) chromatic stimuli are processed by a colour selective pathway separate from that which processes luminance motion. However, at faster speeds colour and luminance motion stimuli are processed by a single pathway that does not signal the colour properties of the stimulus [48-50]. In the light of this potential segregation between colour and luminance motion processing in our first experiment we examined the variation of colour and luminance motion-onset VEPs across the human scalp for a range of stimulus speeds spanning this transition between colour selective and non-colour selective processing mechanisms.

**Fig. (2):**
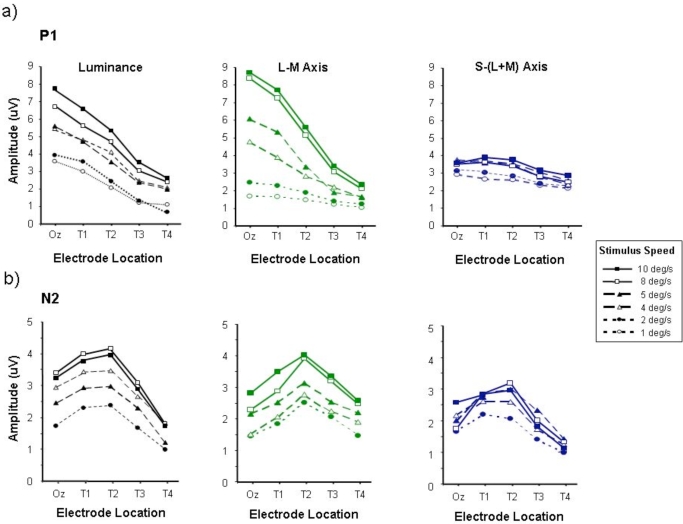
P1 **(a)** and N2 **(b)** amplitudes measured from motion-onset VEPs elicited by luminance, L-M and S/(L+M) chromatic stimuli, plotted as a function of electrode placement. This graph, like all the following, represents averages taken from 6 observers. Data are shown for high contrast stimuli of speeds ranging from 1 - 10 deg/sec. Average standard error approximated to twice the size of the data points.

Fig. (**2**) shows a series of graphs which plot the variation of P1 (Fig. **2a**) and N2 (Fig. **2b**) amplitudes as a function of lateral electrode position for responses elicited by motion onset stimuli with speeds ranging from 1 - 10 deg/s. Data are presented for achromatic (luminance) motion stimuli, as well as for L-M (ø = 0-180) and S-(L+M) (ø = 90-270) chromatic stimuli. The amplitudes of the P1 waves generated by the achromatic and chromatic L-M stimuli appear to be greatest with faster speeds, consistent with previous findings [8]. By comparison, the S-cone isolating stimuli elicit much smaller P1 amplitudes. Another key feature of the P1 wave, particularly when it is elicited by either luminance-defined or L/M chromatically defined motion, is that it is consistently largest for the Oz electrode position located at the occipital pole. Responses recorded from more lateral electrode locations (T1-T4) show an approximately linear decrease in P1 amplitude with increasing distance from Oz which is particularly evident for the faster stimuli (> 4 deg/s). The variation of N2 amplitude with lateral electrode position is shown in Fig. (**2b**). For all three kinds of moving stimuli (luminance, L/M and S/(L+M)) the N2 wave exhibits a different profile to that of the P1 wave in that maximum response amplitude is obtained at a more lateral electrode placement, usually electrode T2. This tendency for the N2 amplitude to peak at lateral electrode positions occurs across the entire range of stimulus speeds tested.

The fact that the motion specific N2 component shows a similar pattern of variation as a function of electrode position for both chromatic and luminance stimuli suggests that the response is generated in similar cortical areas for both types of stimuli. However, one criticism that is often levelled at isoluminant stimuli, particularly large spatially extensive stimuli, is that they may not be entirely free from luminance contrast cues. These intrusions can arise from a number of sources; they may be simply due to inaccuracies in the isoluminant settings by the observers [51] or due to physiological variations in the isoluminant points of ganglion cells at more peripheral retinal locations [52]. With these potential sources of intrusion from luminance mechanisms in mind in a series of control experiments we examined motion-onset VEPs generated by chromatic stimuli the luminance ratios of which were systematically shifted away from the subjective isoluminant settings. This was in order to examine whether precise setting of isoluminance was crucial to the variation of the chromatic motion onset response across the scalp. The results from this experiment are shown in Fig. (**3**). Data are shown for three stimulus speeds 10, 5 and 2 deg/s in which the chromatic stimuli (either L/M or S/(L+M)) were offset from the individual’s isoluminant point by setting the luminance ratio at either +/- 0.1 or +/- 0.05 away from that point. P1 and N2 amplitude vary in a similar fashion to that observed in the previous experiment, with P1 being maximum at Oz and decreasing with increasing laterality whilst N2 is greatest at the more lateral electrode position T2. The small departures from isoluminance do not appear to induce major departures from this pattern. The possible exception to this however, can be observed for the S/(L+M) stimuli at the fastest stimulus speed where the introduction of luminance contrast, whilst not affecting the location (at T2) of the largest N2 response certainly leads to the generation of larger amplitude responses.

### Topographical Variation of the Motion-Onset VEP as a Function of Stimulus Contrast

The contrast of chromatic motion stimuli forms another important parameter which can determine the nature of the underlying motion processing mechanisms that are involved in the analysis of such stimuli. High contrast chromatic stimuli are more likely to be processed by chromatically sensitive low-level motion energy detectors whilst low contrast stimuli are likely to involve more complex analysis and the involvement of ‘higher order’ processes such as position or feature tracking [37-39]. We wished to examine whether the topographical profiles of the P1 and N2 components of the motion-onset VEP would be influenced by varying the contrast of the stimuli in order to ascertain whether the psychophysically derived dichotomy between high and low contrast chromatic motion processing has an electrophysiological correlate. To this end we recorded chromatic and luminance motion-onset VEPs to a range of relatively high and low contrast stimuli.

Fig. (**4a-c**) shows data for motion-onset VEPs elicited by luminance, L/M and S/(L+M) grating stimuli of speeds equal to 10, 5 and 2 deg/s over a range of stimulus contrasts. The contrast range shown for each stimulus type represents that over which discernable responses, comprising P1-N2-P2 triphasic waveforms, could be elicited across all subjects. For the L/M chromatic stimuli the contrast range is particularly limited due to the large spectral overlap between the L- and M-cones. Whilst for the S/(L+M) chromatic stimuli even for relatively high cone contrasts it was in many cases difficult to elicit measurable motion-onset VEPs. Nonetheless over the range of contrasts employed, the topographical variation of the P1 and N2 responses in the luminance and chromatic responses appears similar to that described in the velocity experiments. P1 exhibits a linear decrease in amplitude with increasingly more lateral electrode placements and this occurs for both high and low contrast stimuli. However, once again the P1 responses elicited by the S/(L+M) motion stimuli are of much smaller amplitude and tend not to exhibit a large reduction in amplitude with lateral electrode placement as observed for the luminance and L/M chromatic responses. The amplitude of N2 for the luminance and L/M chromatic stimuli continues to exhibit a maximum for electrode positions that lie lateral to Oz for all speeds and contrasts. The N2 component of the S/(L+M) motion-onset VEP shows a similar topographical distribution except for the slowest stimulus speed tested (2 deg/sec) where the low contrast responses are barely discernable from noise.

## DISCUSSION

The main finding of this study is that motion-onset VEPs generated by luminance and chromatic motion exhibit similar variations in the morphology of their main response components across the human scalp. Measurements of P1 amplitudes elicited by moving chromatic stimuli show that they reach a maximum at electrode position Oz, close to the occipital pole, and decrease with increasingly more lateral electrode locations. The behaviour of P1 in the chromatic response is similar to that shown here and in previous studies for the motion-onset VEP elicited by luminance motion stimuli [5,15,17]. This similarity suggests that the P1 component generated by chromatic motion stimuli, like its luminance counterpart, originates in the striate cortex (V1) and is a reflection of pattern-related processing [4,8,11,16,21]. The similarity between chromatic and luminance responses also extends to the other major component of the motion-onset response, N2. Under all conditions N2 amplitudes for the luminance and chromatic motion stimuli peaked at more lateral electrode positions relative to Oz. This finding is consistent with previous studies which employed luminance motion stimuli and showed N2 to have maximum amplitude at recording sites overlying the extra-striate cortex in the occipito-temporal-parietal regions of the brain [4-8,13,15]. Source localisation studies have also placed the dipole responsible for the generation of the N2 component in a similar region of the extra-striate cortex, in area V5/MT [20,21,53] an area closely identified with motion processing in the human brain [18,19].

### A Common Physiological Pathway for Luminance- and Chromatically-Defined Motion?

A number of psychophysical studies have pointed to the existence of separable low-level processing pathways for colour and luminance motion stimuli [see 54 for a review]. However, the similarity between the topographical variations of motion-onset VEPs generated by luminance and colour points to their generation in similar, if not coincident, regions of the cortex. The correspondence between the behaviour of the colour and luminance responses across the human scalp indicates that whilst there may be initial segregation of colour and luminance motion inputs to the visual system, there is likely to be a high convergence into a common motion pathway further along the processing hierarchy [26,29,35]. This view agrees with previous studies in which responses to isoluminant chromatic motion have been clearly demonstrated in monkey [31] and human V5/MT [36,55]. In macaque area V5/MT approximately a third of the neural population exhibited attenuated responses at isoluminance (both for stimuli modulated along the L/M and S/(L+M) colour axes) but were not completely silenced [32-34]. However, others have found that monkey V5/MT cells are capable of making directionally specific responses at isoluminance, although the responses are less vigorous than for luminance stimuli [31,56]. Other evidence has suggested that chromatic motion maybe analysed by the visual system using a ‘frequency doubled’ response arising from the non-colour selective neurons of the magnocellular system [57]. These responses allow a change in colour to be signalled but without retaining information about the identity of the colours (i.e. it is unsigned). Dobkins and Albright [32] confirmed that this is the case with recordings from monkey MT, as they found that direction of motion is perceived in the direction of the nearest chromatic border regardless of whether the colour (or sign) remained constant or reversed. However, they also found that when the direction of motion signalled by chromatic borders is ambiguous, the visual system is capable of perceiving direction of motion by following components of the same colour/sign. Taken together, the evidence points to the likelihood that neurons in V5/MT are capable of processing motion information regardless of whether the motion is defined by luminance or colour (see also [58,59]). Other regions may also be involved in the processing of chromatic motion signals. Area V3 is another extra-striate area considered to form part of the cortical motion-processing pathway. Indeed, contributions from areas V3/V3a to the generation of the motion related VEP have been previously suggested [60,61]. Studies of V3 cells in the macaque found that between 40-60% displayed direction selectivity, and the majority of the cells that were tested with varying speeds, were tuned for speed with peak responses in the population ranging mainly from 4-32 ^0^/sec. However, despite earlier claims of an absence of colour processing cells in V3, colour selective cells were also observed (including a large number with S-cone input) with a significant proportion of cells exhibiting both directional and chromatic tuning [62,63]. Connections between V3 and V4 have also been found suggesting that V3 is in fact part of both the motion and colour pathways [64,65].

Whilst the foregoing discussion emphasizes the evidence for a common anatomical and physiological processing pathway for the analysis of motion defined by colour and luminance stimuli, other studies point to the existence of separable colour and luminance processing mechanisms rather than one which simply combines inputs deriving from the two kinds of motion stimuli [37-40,66-68]. Our VEP data, however, fail to demonstrate major differences between responses elicited by motion defined by colour and luminance. This may be due to a number of reasons. One possibility is that whilst colour and luminance motion might be processed in the same cortical regions, namely area V5/MT, it might be undertaken by different sub-populations of neurons. The spatial proximity of these neurons in area V5/MT would mean that their responses would be unlikely to be differentiated by the rather coarse topographical sampling achieved by the electrode montage used in this study. However, evidence from single unit studies in the macaque indicates that V5 neurons exhibit correlated thresholds for colour and luminance motion. This suggests that neurons in V5/MT exhibit cue invariant responses to both colour and luminance motion rather than comprising two separate sub-populations responsive to either one or the other type of motion stimulus [56]. Another reason for the failure of our data to show differences between the behaviour of colour and luminance motion-onset VEPs might lie in the fact that certain studies suggest that the separability of colour and luminance motion processing is dependent upon particular stimulus parameters. Stimulus contrast, for example, is one aspect that appears to be critical in determining whether chromatic motion is processed by low-level motion processing mechanisms, similar to luminance motion, or whether it is processed by separate higher level or feature tracking mechanisms. High contrast chromatic stimuli are processed by the former type, whilst those of low contrast are processed by the latter [37-41]. It may simply be due to the fact that the low contrast chromatic stimuli used in this study were simply not of low enough contrast to ensure the involvement of the ‘higher order’ chromatic mechanisms that are distinct from the low-level processes that also are involved in luminance motion processing. The motion-onset VEP may be an unsuitable method for eliciting responses from stimuli that are processed by higher order processing mechanisms because of the problems of signal to noise ratios inherent in the use of low contrast stimuli.

A persistent feature of the results in this study is the low amplitude motion-onset VEPs that are obtained for stimuli which isolate S-cone mechanisms in comparison to those elicited by luminance and L/M cone isolating stimuli. This relative deficiency of the S-cone mediated motion responses is consistent with the nature of cone inputs to V5/MT that have been described at the single neuronal level. These studies have demonstrated that S-cone input to V5/MT is very much weaker than that from L- and M-cones by a factor of almost 10 times [56]. Other single unit and neuroimaging studies have also emphasized the paucity of S-cone input to the motion related processing regions of the cortex [34,36]. So whilst there is good evidence for the existence of S-cone representation in V5/MT which is sufficient to support the perception of chromatic motion it is not at the same level of L- and M-cone representation, hence the reason why tritan or S-cone isolating motion stimuli fail to elicit prominent motion-onset VEPs. However, previous studies have demonstrated there is a close degree of similarity between the pattern of responses generated by tritan and luminance motion stimuli [69].

In summary, we have shown that motion-onset VEPs elicited by colour and luminance motion stimuli exhibit qualitatively similar patterns of variation across the human scalp - suggesting similar origins in the brain for the different components of the responses. Furthermore, the differences reported here between the topographical variation of the P1 and N2 components of the chromatic and luminance motion-onset VEPs, are consistent with the notion that these components have different cortical sites of generation and reflect neural activity involved in different aspects of motion processing [4-8].

## Figures and Tables

**Fig. (1):**
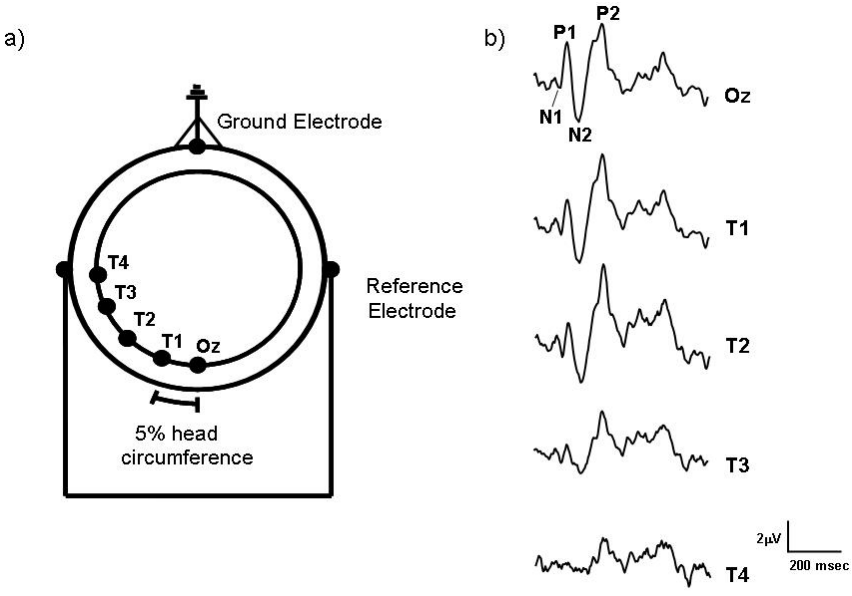
**(a)** The electrode montage used to record motion-onset VEPs from the human scalp. Starting from Oz, each of the lateral electrodes (T1-T4) were placed at intervals equal to 5% of the observers’ head circumference. The ground electrode was placed on the forehead and linked ear electrodes acted as reference. **(b)** Sample motion-onset VEPs recorded from a single subject which were elicited by a moving high contrast L-M chromatic stimulus (10 deg/s) recorded from the five different electrode sites (Oz - T4).

**Fig. (3):**
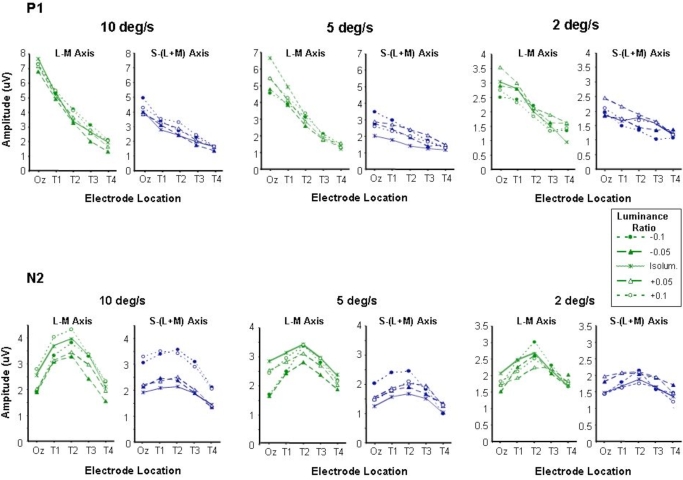
P1 **(a)** and N2 **(b)** amplitudes plotted as a function of electrode location for moving chromatic stimuli of different luminance ratios. Small amounts of luminance contrast were introduced into chromatic stimuli by shifting the luminance ratio (up to +/- 0.1) away from the individually set isoluminant point. This was done for both L-M and S-(L+M) chromatic stimuli at speeds of 10, 5 and 2 deg/sec.

**Fig. (4):**
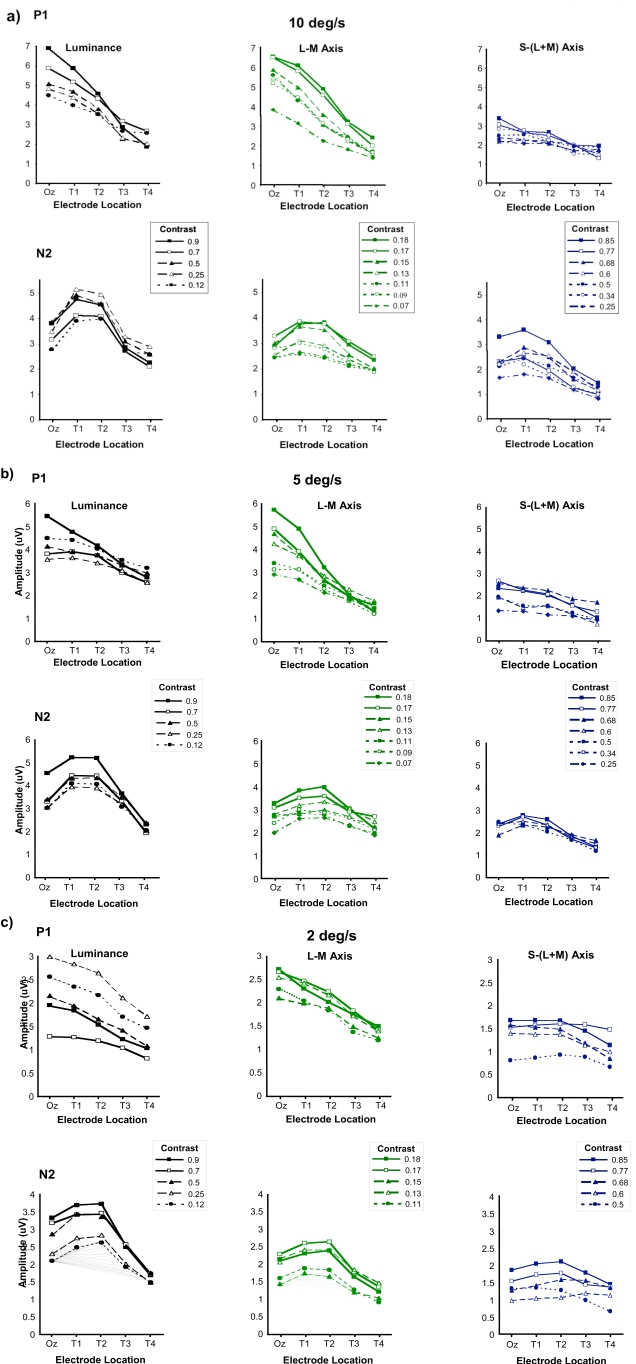
P1 and N2 amplitudes plotted as a function of electrode location for luminance, L-M and S/(L+M) stimuli of varying contrast. The contrast values for the luminance stimuli are in terms of Michelson contrast, those for the L-M and S-(L+M) chromatic stimuli are given in terms of a computed cone contrast value (see text for details). Data are shown for moving stimuli of relatively high (10 deg/sec), medium (5 deg/sec) and low (2 deg/sec) speed.
